# An Analysis of Orthopaedic Job Trends in the United States Over the Past 30 years

**DOI:** 10.5435/JAAOSGlobal-D-17-00056

**Published:** 2018-08-17

**Authors:** Sandeep Mannava, Alexander H. Jinnah, Mark E. Cinque, Johannes F. Plate, Riyaz H. Jinnah, Robert F. LaPrade, David F. Martin, L. Andrew Koman

**Affiliations:** From the American Board of Medical Specialties (ABMS)—American Board of Orthopaedic Surgery (ABOS) visiting scholar at the Steadman Philippon Research Institute, Vail, CO (Dr. Mannava); the Department of Orthopaedic Surgery, Wake Forest University School of Medicine, Medical Center Boulevard, Winston-Salem, NC (Dr. A. H. Jinnah, Dr. Plate, Dr. R. H. Jinnah, and Dr. Koman); the Steadman Philippon Research Institute, Vail, CO (Dr. Cinque, Dr. LaPrade); and American Board of Orthopaedic Surgery (ABOS), Chapel Hill, NC (Dr. Martin).

## Abstract

**Introduction:**

Orthopaedic surgery residency training requires 5 clinical years; fellowship subspecialty training requires an additional year. Orthopaedic surgery fellowship training has financial implications regarding potential career earnings and opportunity cost. To evaluate the effect of fellowship training on employment, 30 years of orthopaedic job advertisements were analyzed to determine fellowship requirements for academic centers, private practices, urban areas, and rural areas. It was hypothesized that subspecialty training is an important prerequisite for orthopaedic employment.

**Methods:**

Job advertisements in the *Journal of Bone and Joint Surgery* (*JBJS Am*) and *Orthopedics* were analyzed to determine whether fellowship training versus “generalist” (no subspecialty fellowship) positions were advertised for the years 1984, 1989, 1994, 1999, 2004, 2009, and 2014. Jobs were categorized as academic (defined by the requirement to teach medical students, residents, or fellows); private practice; rural (defined as population under 200,000); and urban. “General” orthopaedic surgery job postings were defined as job advertisements that did not require fellowship training.

**Results:**

A total of 4,720 job advertisements were analyzed. From 1984 to 2014, the percentage of advertised jobs requiring fellowship training increased from 5% to 68% (*P* < 0.05). Conversely, from 1984 to 2014, the percentage of advertised jobs targeting general orthopaedic surgeons decreased from 95% to 32% (*P* < 0.05). Between 2009 and 2014, advertised jobs requiring fellowship surpassed general orthopaedic surgery jobs.

**Conclusions:**

Over the past 30 years, there was a trend toward fellowship being required as part of the advertised orthopaedic jobs available to graduates of orthopaedic training programs. The reasons for increased orthopaedic training are likely multifactorial, including limited clinical duty hours during orthopaedic residency, advertisement and marketing forces emphasizing super-sub-specialty care in multispecialty orthopaedic groups, and the greater complexity of orthopaedic procedures being performed.

Most orthopaedic surgery residents elect additional subspecialty fellowship training. To evaluate the effect of fellowship training on employment, 30 years of orthopaedic job advertisements were analyzed to determine fellowship requirements for academic centers, private practices, urban areas, and rural areas. It was hypothesized that subspecialty training is an important prerequisite for orthopaedic employment.

## Methods

Job advertisements in the *Journal of Bone and Joint Surgery* (*JBJS Am*) and *Orthopedics* were analyzed to determine whether fellowship training versus “generalist” (no subspecialty fellowship) positions were advertised for the years 1984, 1989, 1994, 1999, 2004, 2009, and 2014. Jobs were categorized as academic (defined by the requirement to teach medical students, residents, or fellows); private practice; rural (defined as population under 200,000); and urban. “General” orthopaedic surgery job postings were defined as job advertisements that did not require fellowship training. The Cochran-Armitage trend test and Poisson regression modeling were used for statistical analysis. All analyses were performed in SAS Version 9.4, and a 0.05 significance level was used throughout this analysis.

## Results

**Figure 1:**
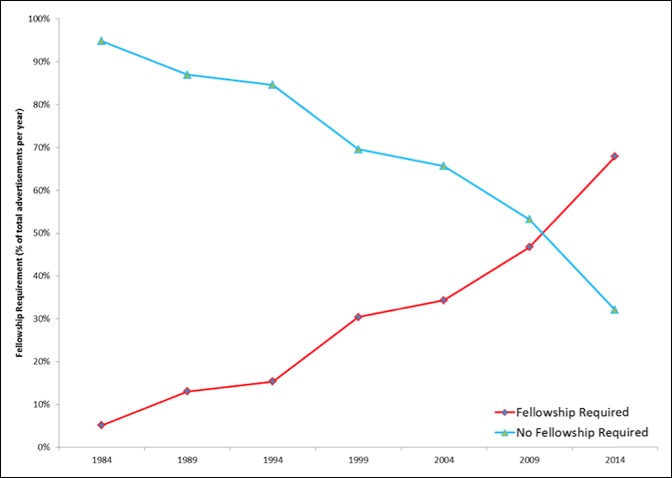
Graph showing analysis of advertised orthopaedic surgery jobs: advertised orthopaedic surgery jobs requiring fellowship training increased from 5% in 1984 to 68% in 2014 (*P* < 0.05). Between 2009 and 2014, the number of advertised jobs requiring fellowship surpassed general orthopaedic surgery jobs.

A total of 4,720 job advertisements were analyzed. From 1984 to 2014, the percentage of advertised jobs requiring fellowship training increased from 5% to 68% (Figure 1; *P* < 0.05). Conversely, from 1984 to 2014, the percentage of advertised jobs targeting general orthopaedic surgeons decreased from 95% to 32% (Figure 1; *P* < 0.05). Between 2009 and 2014, advertised jobs requiring fellowship surpassed general orthopaedic surgery jobs (Figure 1).

**Figure 2:**
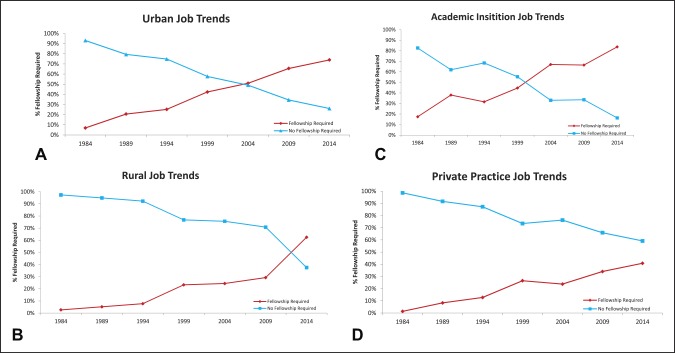
Graphs showing analysis of specific advertised orthopaedic surgery job markets: Between 1999 and 2014, the number of job advertisements requiring fellowship training surpassed advertised general orthopaedic surgery jobs in the urban, rural, and academic practice setting. **A**, Advertised orthopaedic jobs requiring fellowship in urban areas increased from 7% in 1984 to 74% in 2014 (*P* < 0.05). **B**, In rural areas, the fellowship requirements in advertised jobs increased from 3% to 63% over the 30-year study period (*P* < 0.05). **C**, The advertised orthopaedic positions requiring fellowship in academic centers increased from 17% in 1984 to 84% in 2014 (*P* < 0.05). **D**, Furthermore, private practice job advertisements requiring fellowships increased from 1% in 1984 to 41% in 2014 (*P* < 0.05). Advertisements for private practice orthopaedic surgery jobs demonstrated preference for general orthopaedic surgeons or no fellowship training throughout the 30-year study period.

In 1984, 6.9% of advertised urban jobs and 2.6% of advertised rural jobs required fellowship training; this increased by 2014 to 74% of urban jobs (Figure 2, A; *P* < 0.05) and 62.5% of rural jobs (Figure 2, B; *P* < 0.05). In 1984, 17.4% of advertised academic jobs and 1.4% of advertised nonacademic jobs required fellowship training; this increased by 2014 to 83.9% of advertised academic jobs (Figure 2, C; *P* < 0.05) and 41.1% of advertised nonacademic jobs requiring fellowship training (Figure 2, D; *P* < 0.05). Between 1999 and 2014, the job advertisements requiring fellowship training surpassed “generalist” positions in the urban, rural, and academic practice setting (Figure 2, A–C). Advertisements for private practice orthopaedic surgery jobs demonstrated preference for general orthopaedic surgeons throughout the 30-year study period (Figure 2, D).

## Conclusions

Orthopaedic surgery residency training requires five clinical years; fellowship subspecialty training requires an additional year. Orthopaedic surgery fellowship training has financial implications regarding potential career earnings and opportunity cost.^1^ Despite the fact that fellowships often cost residents more than they earn, >90% of residents now complete fellowships.^2^ Horst et al^2^ reported on data from the American Board of Orthopaedic Surgery (ABOS) part 2 certifying examination database, “generalists” decreased from 44.2% to 28.7% from 1990 to 2006. Moreover, from 2003 to 2013, fellowship-trained applicants taking American Board of Orthopaedic Surgery part 2 increased from 76% to 90%.^2^

A limitation of the current study is that only two sources of orthopaedic surgery job advertisements were analyzed. Our analysis allows for consistent comparative methodology over the 30-year study period in two long-standing general orthopaedic journals. Online advertisements were not as prevalent in 1984 and would complicate analysis of trends over the study period. Furthermore, we did not delineate nonacademic positions into private groups versus hospital employed, but we speculate that these followed the overall trends seen.

Over the past 30 years, there was a trend toward fellowship being required as part of the advertised orthopaedic jobs available to graduates of orthopaedic training programs.^3^ The reasons for increased orthopaedic training are likely multifactorial, including limited clinical duty hours during orthopaedic residency,^4,5^ advertisement and marketing forces emphasizing super-sub-specialty care in multispecialty orthopaedic groups,^6^ and the greater complexity of orthopaedic procedures being performed.
